# Open-source low-cost non-contact ECG monitoring system using active dry electrodes

**DOI:** 10.1016/j.ohx.2025.e00718

**Published:** 2025-10-27

**Authors:** Siluo Chen, Yu Chen, Jing Huang, Chengyu Liu, Kan Luo

**Affiliations:** a School of Electronic, Electrical Engineering and Physics, Fujian University of Technology, Fuzhou 350118, China; b Fuzhou Industrial Integration Automation Technology Innovation Center, Fuzhou 350118, China; c State Key Laboratory of Digital Medical Engineering, School of Instrument Science and Engineering, Southeast University, Nanjing 210096, China

**Keywords:** Non-contact ECG, Wearable device, Active dry electrode, Capacitive coupling, Analog front-end, Open-source hardware

## Abstract

Continuous electrocardiogram (ECG) monitoring is essential for the early detection of cardiac arrhythmias and other heart conditions. However, conventional wet electrode-based systems often cause skin irritation and signal degradation, making them uncomfortable and unsuitable for long-term daily use. In this study, we present a fully open-source, low-cost (∼$14), miniaturized (∼50 × 35 × 28 mm^3^), and lightweight (∼60 g), wearable ECG monitoring platform that uses non-contact, active, dry electrodes based on capacitive coupling. The system integrates ultra-high input impedance electrode circuitry, an analog front-end integrated circuit, Bluetooth wireless communication, and an Android mobile application for real-time signal visualization and storage. Comprehensive validation shows that the system reliably acquires ECG signals through light to moderate clothing, with clear preservation of key features, such as QRS complexes, and accurate heart rate estimation. Comparative experiments with a commercial, clinical-grade ECG monitor demonstrate excellent agreement in rhythm tracking, and systematic module-level power analysis highlights further opportunities for low-power optimization. All hardware designs, firmware, and software are openly released to accelerate innovation and reproducibility in the field of wearable, non-contact ECG monitoring. The proposed platform provides a practical, extensible, and accessible reference for researchers aiming to advance next-generation cardiac monitoring technologies.

## Graphical abstract

**Figure:**
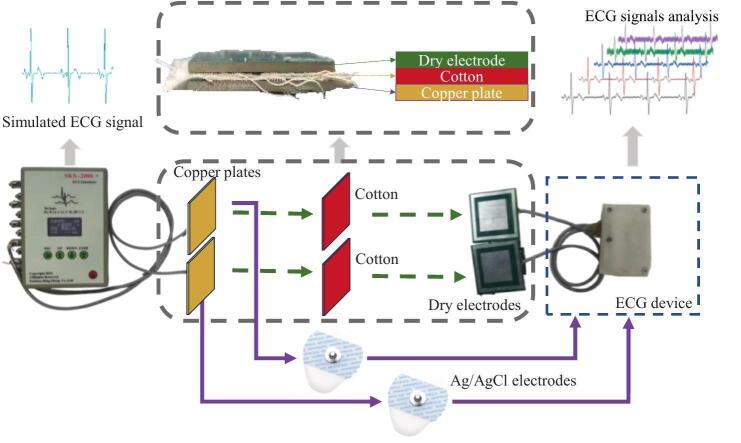

Specifications table

**Table:** 

Hardware name	Non-contact electrocardiogram monitoring system
Subject area	• Engineering and materials science • Medical (e.g., pharmaceutical science) • Educational tools and open source alternatives to existing infrastructure
Hardware type	• Measuring physical properties and in-lab sensors • Field measurements and sensors • Electrical engineering and computer science • Mechanical engineering and materials science
Closest commercial analog	Commercial wearable ECG monitors such as the AliveCor KardiaMobile, or traditional Holter monitors. However, unlike these, our device supports non-contact ECG acquisition and is designed for low-cost.
Open source license	MIT License
Cost of hardware	∼ USD 14
Source file repository	https://zenodo.org/records/15765815

## 1 Hardware in context

Electrocardiography (ECG) is widely regarded as one of the most practical diagnostic tools in clinical medicine. It provides accurate insights into both the physiological and pathological characteristics of cardiac activity. It is routinely used to detect arrhythmias, myocardial infarction, and a variety of other heart diseases [1,2]. However, traditional ECG monitoring is usually performed by trained personnel in clinical settings and involves short measurement durations. This approach is insufficient for detecting infrequent cardiac events. In response to the rapidly growing demand for continuous and long-term ECG monitoring, there has been increasing research interest in developing miniaturized, wearable, highly comfortable ECG monitoring systems [3]. The development of electrodes that can deliver high-quality potential measurements while maintaining user comfort and a lightweight design has become a primary focus.

As the main interface for signal acquisition, electrodes play a decisive role in the overall performance of ECG monitoring systems [4]. Currently, wet Ag/AgCl electrodes are the most commonly used type in clinical practice. However, these electrodes rely on a gel interface that is non-breathable and tends to dry out over time. This results in a decline in signal quality and an increased risk of skin irritation or even ulceration during prolonged use [5,6]. These limitations render wet electrodes unsuitable for long-term or continuous daily monitoring. In response, researchers have developed novel wet electrodes made of composite materials and fabricated using advanced techniques to improve stretchability, self-healing, and biocompatibility [[7], [8], [9]]. Despite these advances, persistent issues such as gel drying and skin irritation remain unresolved, prompting a growing shift toward the development of dry electrodes.

Research in the development of dry electrodes has expanded beyond traditional direct-contact designs, such as conductive fabric [10] and metal electrodes [11], to include flexible dry electrodes fabricated from advanced materials and processes. These processes include thin-film metals deposited on polymer substrates and silver nanoparticle inks applied by inkjet printing [12,13]. These innovations aim to provide excellent electrical conductivity, mechanical flexibility, and improved wearing comfort. Another significant trend in dry electrode development is the adoption of non-contact solutions, particularly capacitive electrodes, which allow for ECG acquisition without direct skin contact [14]. These electrodes use capacitive coupling to detect subtle surface potentials even through clothing, significantly enhancing user comfort and enabling a wider range of ECG monitoring scenarios [15]. Overall, advances in dry electrode technology, particularly those using flexible substrates and novel materials, are essential for creating miniaturized, comfortable, and truly wearable ECG monitoring systems.

Further innovation in ECG hardware design is essential to achieve long-term, continuous, and even non-contact cardiac monitoring. Key requirements include lightweight, compact device structures, ease of wear, and especially high-input-impedance analog front-end (AFE) circuits to maintain optimal signal quality. Although multi-lead wearable systems using silicone dry electrodes have been developed for comprehensive cardiac monitoring [16], their inherent complexity and bulkiness limit their suitability for daily, long-term daily use. Consequently, there is increasing interest in simpler, more compact wearable solutions, some of which have been optimized to weigh as little as 23 g [17]. This level of miniaturization allows for more comfortable and discreet wearing positions than traditional sites like the waist or chest without significantly compromising signal quality [18]. An additional challenge in non-contact ECG monitoring lies in designing and optimizing the AFE circuitry. Recent research efforts have focused on improving amplifier input impedance through techniques such as negative impedance converters and power bootstrapping. These techniques enable the effective detection of weak bioelectrical signals via non-contact dry electrodes [19,20]. Moreover, significant progress has also been made in developing robust noise immunity and anomaly detection algorithms [21,22].

In this study, we present a fully open-source, low-cost, and miniaturized wearable ECG monitoring system designed for non-contact capacitive-coupled dry electrodes. The system integrates high-input-impedance AFE circuitry, Bluetooth wireless transmission, and a user-friendly mobile app that enables real-time visualization and data storage. By openly sharing all of our hardware designs, firmware, and software resources, we aim to lower technical barriers and promote further innovation in wearable non-contact ECG technology.

## 2 Hardware description

**Fig. 1:**
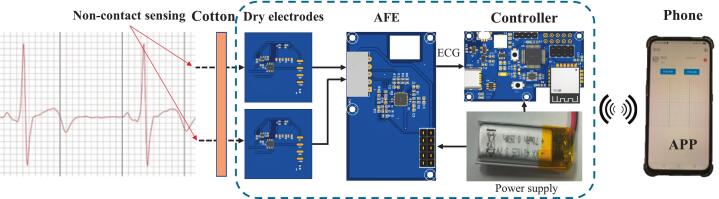
System diagram of the non-contact ECG system.

Fig. 1 shows the system diagram of the proposed non-contact ECG measurement device. As shown within the dashed box, the system consists primarily of the following functional modules, from left to right: non-contact dry electrodes, an AFE board, a device controller with Bluetooth communication capabilities, and a lithium battery.

Non-contact dry electrodes capture surface biopotentials from the skin using the principle of capacitive coupling. These electrodes can detect subtle cardiac signals through a thin insulating layer, thus eliminating the need for conductive gels or direct skin contact. Each dry electrode operates as an active electrode and incorporates an operational amplifier circuit to achieve ultra-high input impedance. This helps suppress noise and mitigate the effects of parasitic capacitance. The differential biopotential signals detected by the two electrodes are then transmitted to the AFE module for further amplification and hardware filtering. This stage effectively amplifies and filters the weak ECG signals, resulting in improved signal quality and reduced noise. The conditioned signals are sent to the device controller, which digitizes the analog signals and manages system functions, including power management and wireless communication. Finally, the processed ECG data is transmitted via Bluetooth to a mobile phone for real-time display, analysis, and storage.

### 2.1 Non-contact active dry electrodes

The active dry electrode is the most critical component of our non-contact ECG system. Non-contact measurement typically involves high total equivalent impedance at the skin-electrode interface [23], and ECG signals are inherently low-frequency and weak in amplitude [24]. To address these challenges, the electrodes are designed to acquire signals via capacitive coupling, which necessitates the use of active electrodes with ultra-high input impedance. This ensures proper impedance matching, enabling the effective capture of subtle surface biopotentials.

**Fig. 2:**
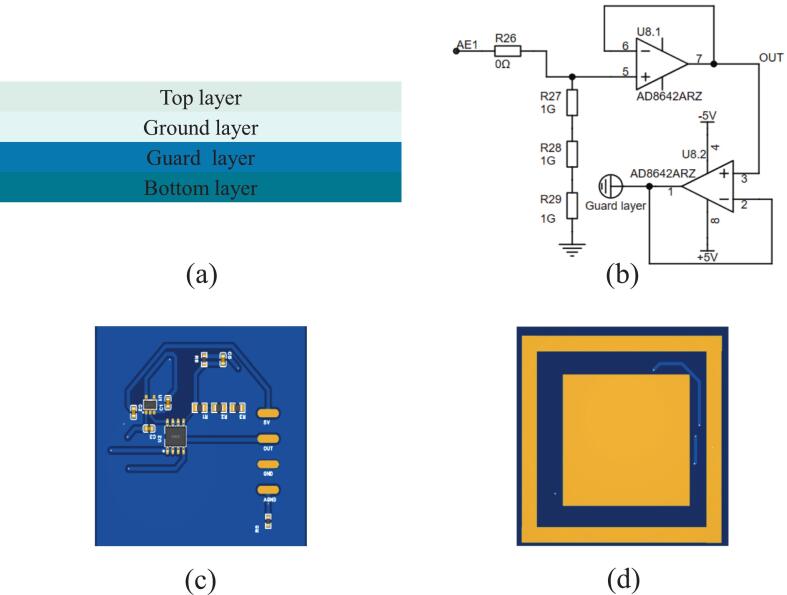
Non-contact active dry electrode design. (a) Layered structure of the electrode. (b) Schematic of the active electrode circuit based on a high-input-impedance amplifier. (c) and (d) show the front and back views of the electrode’s PCB layout.

Fig. 2 shows the design of the non-contact active dry electrode. As illustrated in Fig. 2(a), the electrode has four layers: the top, ground, guard, and bottom. The top and ground layers primarily implement the active electrode circuitry, and the bottom layer serves as the sensing surface. The guard layer is designed for noise suppression to minimize interference. To achieve ultra-high input impedance, the electrode circuit integrates a high-input-impedance operational amplifier, as shown in Fig. 2(b). Following the approach described in references [25,26], the active electrode uses AD8642 amplifiers (Analog Devices, Norwood, MA, USA). The AD8642 contains two operational amplifiers, both of which are configured as voltage followers. The first amplifier provides high input impedance and low output impedance to minimize signal loss between the capacitive sensing layer and the subsequent AFE. The second amplifier drives the guard layer, reducing parasitic capacitance and enhancing noise immunity. The amplifiers are powered by ± 5 V from the TPS60403 (Texas Instruments). Fig. 2 (c) and (d) show the front and back views of the electrode’s printed circuit board (PCB) layout. The top layer contains electronic components, and the bottom layer contains the capacitive sensing structure. The overall PCB dimensions are 50 × 50 mm^2^, with the central capacitive sensing plate measuring 30 × 30 mm^2^. A guard ring with a width of 3.5 mm surrounds it and is electrically connected to the guard layer to provide shielding and reduce noise interference.

### 2.2 Analog front-end board

The ECG signals transmitted from the active dry electrodes are still relatively weak and noisy and require further amplification and filtering. Compared to traditional multi-stage amplification and filtering circuits built with discrete components, using an integrated AFE chip offers a more compact and efficient solution [27]. In our design, we use the AD8232 (SparkFun LLC, Niwot, Colorado) [28] for signal conditioning.

**Fig. 3:**
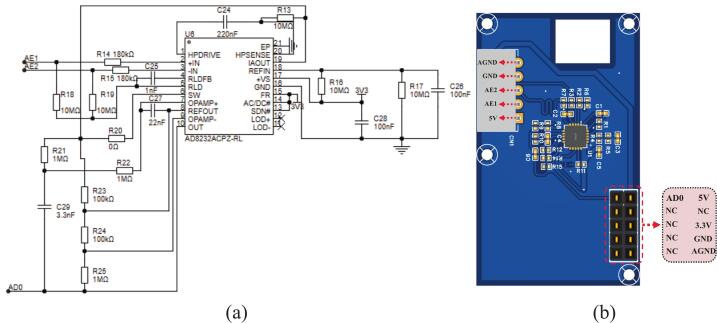
AFE board design. (a) Schematic of the AD8232;(b) PCB layout of the AFE board.

The AD8232 provides a high common-mode rejection ratio of up to 80 dB. It can be configured as a bipolar high-pass filter, which suppresses motion artifacts and baseline drift. This ensures that the signal quality meets the requirements for ECG monitoring. The detailed circuit design is shown in Fig. 3(a). Since the system is designed with a dual-electrode configuration, two 10 MΩ bias resistors are connected to pin 5 of the AD8232, each is linked to one of the differential input channels, enabling common-mode rejection. Since the QRS complex is closely related to arrhythmia detection and primarily lies within the 8–20 Hz frequency range [29], this design focuses on capturing those components. According to the AD8232 datasheet [30], the circuit includes a 7 Hz high-pass filter and a 24 Hz low-pass filter to suppress baseline wander, motion artifacts, and high-frequency interference. The amplified and filtered signal from the AD8232 is output through pin 10 of the chip and routed to the controller’s ADC input pin PA0, which is labeled AD0. AE1 and AE2 are connected to the two dry electrodes, respectively. All other configurations follow the recommendations in the AD8232 datasheet. The PCB layout of the AFE board is shown in Fig. 3(b).

### 2.3 Main controller board

**Fig. 4:**
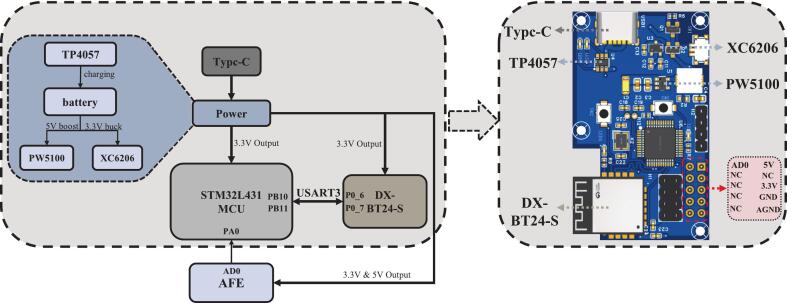
Main controller board design.

Fig. 4 shows the design of the main controller board, including the system block diagram and PCB layout. This board serves as the core processing and communication unit of the non-contact ECG monitoring system. It integrates signal acquisition, data processing, Bluetooth communication, and power management.

The 32-bit STM32L431CCT6 microcontroller (STMicroelectronics) is at the heart of the controller board. It was selected for its low power consumption and efficient processing capability. It receives analog ECG signals from the AFE via net AD0, connected to pin PA0, and digitizes them using its built-in ADC, following the approach in [31]. Considering the primary ECG signal bandwidth of 1–40 Hz for ambulatory monitoring [25], we apply a 5 × oversampling strategy. According to the Nyquist sampling theorem, the ADC sampling rate is set to 200 Hz. The digitized ECG signals are then processed with digital filtering and then transmitted wirelessly via the BT24-S Bluetooth module (Shenzhen DX-SMART Technology Co., Ltd), which supports the BLE 5.1 protocol with a maximum transmission rate of up to 10 KB/s. The module connects to the MCU via the USART3 interface (PB10 for TX and PB11 for RX) and is controlled through pins P0_6 and P0_9. The system is powered by a lithium battery, and the TP4057 chip manages charging. The PW5100 handles voltage regulation for the 5 V boost, and the XC6206 handles voltage regulation for the 3.3 V buck. These components provide stable power to both the MCU and the Bluetooth module. providing stable power to both the MCU and the Bluetooth module. Circuit schematics of the system are publicly available on Zenodo (https://zenodo.org/records/15765815).

## 3 Design files summary

This section provides an overview of the design files associated with the non-contact ECG device. The mechanical components, including the top cover and enclosure, were designed using SolidWorks, which is a widely used computer-aided design (CAD) software program for creating detailed 3D models. The corresponding files are provided in.STL format. The electronic circuit design was completed using JLCEDA Pro V2.1.57 to create the circuit schematics and layouts. The design files are available in.ePro and.PDF formats. JLCEDA Pro is freely available to individual users and supports rapid electronic design and documentation. The mobile application for ECG signal visualization and user interface interaction was developed using the Android Studio platform. The complete source code is provided as a compressed (.zip) package. Firmware development for the controller was carried out using Keil uVision5 and STM32CubeMX. The embedded source code is also provided in.zip format. A summary of these design files is presented in Table 1. All design files have been uploaded to an open-source platform for public access and use.

**Table 1:** Design file form.

**Folder Name**	**Design file name**	**File type**	**Open source license**
Mechanical design	Lid	.STL	MIT License
	Enclosure	.STL	
Circuit design	TopPcb	.epro,.pdf	MIT License
	BasePcb	.epro,.pdf	
	ElectrodePcb	.epro,.pdf	
Mobile software	EcgMave	.zip	MIT License
Stm32 program	Stm32	.zip	MIT License

## 4 Bill of materials summary

The materials used in the system are primarily categorized into two types: electronic components and mechanical structural components. The electronic components include all circuitry-related elements, such as microcontrollers, power management chips, and communication modules. The mechanical components consist of structural and wearable elements, including the enclosure, copper standoffs, screws, and the chest strap.

Table 2 and Table 3 provide detailed information about these components and offer comprehensive lists of all the electronic and mechanical parts required for device assembly. Each table includes the component name, specifications, quantity, unit price, and total cost, ensuring clarity, transparency, and traceability in material usage and budgeting throughout the system development process. According to the bill of materials, the system has a low-cost design with an approximate total cost of $14.

**Table 2:** Circuit components form.

**Designator**	**Component**	**Number**	**Cost per unit-USD**	**Total cost** **−USD**	**Source of materials**	**Material type**
C1	TAJA226K010RNJ	1	0.07	0.07	https://e.tb.cn/h.6oaaw8pdw883ICK	Non-specific
C2	100nF C0603	1	0.01	0.01	https://e.tb.cn/h.6o2B8T9jINHH9QI	Non-specific
C3	22uF C0603	1	0.01	0.01	https://e.tb.cn/h.6oVpLjEPOz6LyNb	Non-specific
C4	4.7nF C0603	1	0.01	0.01	https://e.tb.cn/h.6oVpLjEPOz6LyNb	Non-specific
C5	10uF C0603	1	0.01	0.01	https://e.tb.cn/h.6oVpLjEPOz6LyNb	Non-specific
C6, C9	10uF C0402	2	0.01	0.02	https://e.tb.cn/h.6odTWhh0b0ldSwo	Non-specific
C7	1uF C0402	1	0.01	0.01	https://e.tb.cn/h.6odTWhh0b0ldSwo	Non-specific
C8, C10, C11, C12, C13, C14, C17, C18, C21, C23, C26, C28	100nF C0402	12	0.01	0.12	https://e.tb.cn/h.6odTWhh0b0ldSwo	Non-specific
C15, C16	6pF C0402	2	0.01	0.02	https://e.tb.cn/h.6odTWhh0b0ldSwo	Non-specific
C19, C22	22pF C0402	2	0.01	0.02	https://e.tb.cn/h.6odTWhh0b0ldSwo	Non-specific
C20	22uF C0402	1	0.01	0.01	https://e.tb.cn/h.6odTWhh0b0ldSwo	Non-specific
C24	220nF C0402	1	0.01	0.01	https://e.tb.cn/h.6odTWhh0b0ldSwo	Non-specific
C25	1nF C0402	1	0.01	0.01	https://e.tb.cn/h.6odTWhh0b0ldSwo	Non-specific
C27	22nF C0402	1	0.01	0.01	https://e.tb.cn/h.6odTWhh0b0ldSwo	Non-specific
C29	3.3nF C0402	1	0.01	0.01	https://e.tb.cn/h.6odTWhh0b0ldSwo	Non-specific
C30, C31, C32	1uF C0603	3	0.01	0.03	https://e.tb.cn/h.6oVpLjEPOz6LyNb	Non-specific
CN1	WF-1F200A2P	1	0.02	0.02	https://e.tb.cn/h.6oaGrtKMj27dYHv	Non-specific
CN2	XH-5AW	1	0.01	0.01	https://e.tb.cn/h.6oaDRO7LF2Fl0tQ	Non-specific
H1	PZ254V-12-8P	1	0.01	0.01	https://e.tb.cn/h.6oZ164qefKnrLfK	Non-specific
H2	PZ254V-11-04P	1	0.01	0.01	https://e.tb.cn/h.6o49dBnfYMma3sg	Non-specific
H3	PZ254V-12-10P	1	0.01	0.01	https://e.tb.cn/h.6o49dBnfYMma3sg	Non-specific
H4	PM254-2–05-Z-8.5	1	0.01	0.01	https://e.tb.cn/h.6oWaViCkWJWwX1b	Non-specific
L1	10uH L0503	1	0.01	0.01	https://e.tb.cn/h.6o4LiQOGgNQ97hU	L0503
LED1, LED2, LED3, LED4	LED0402-RD	4	0.01	0.04	https://e.tb.cn/h.6oUgiO5QqruGixi	Non-specific
Q1, Q2	AO3401A	2	0.02	0.04	https://e.tb.cn/h.6o4DPNww4viUhxv	Non-specific
R1, R4	5.1 K R0402	2	0.01	0.02	https://e.tb.cn/h.6o4zXkGTnEM1MDu	Non-specific
R2	0.5R R0603	1	0.01	0.01	https://e.tb.cn/h.6ofVDgKmLJ5bXzS	Non-specific
R3	240 K R0402	1	0.01	0.01	https://e.tb.cn/h.6o4zXkGTnEM1MDu	Non-specific
R5	33 K R0402	1	0.01	0.01	https://e.tb.cn/h.6o4zXkGTnEM1MDu	Non-specific
R6	3.3 K R0402	1	0.01	0.01	https://e.tb.cn/h.6o4zXkGTnEM1MDu	Non-specific
R7, R9	1 K R0402	2	0.01	0.02	https://e.tb.cn/h.6o4zXkGTnEM1MDu	Non-specific
R8, R23, R24	100 K R0402	3	0.01	0.03	https://e.tb.cn/h.6o4zXkGTnEM1MDu	Non-specific
R10, R11, R12	10 K R0402	3	0.01	0.03	https://e.tb.cn/h.6o4zXkGTnEM1MDu	Non-specific
R13, R16, R17, R18, R19	10 M R0402	5	0.01	0.05	https://e.tb.cn/h.6o4zXkGTnEM1MDu	Non-specific
R14, R15	180 K R0402	2	0.01	0.02	https://e.tb.cn/h.6o4zXkGTnEM1MDu	Non-specific
R20	0R R0402	1	0.01	0.01	https://e.tb.cn/h.6o4zXkGTnEM1MDu	Non-specific
R21, R22, R25	1 M R0402	1	0.01	0.03	https://e.tb.cn/h.6o4zXkGTnEM1MDu	Non-specific
R26, R30	0R R0603	2	0.01	0.04	https://e.tb.cn/h.6oUCKdJQicNKPxB	Non-specific
R27, R28, R29	1G R0603	6	0.12	0.72	https://e.tb.cn/h.6o3aVP6HkNjr9NG	Non-specific
SW1	MINI MSK12CO2	1	0.05	0.05	https://e.tb.cn/h.6o0WLB2Pd5U8Ydz	Non-specific
SW2, SW3	TS-1088-AR02016	2	0.03	0.06	https://e.tb.cn/h.6o5Wak0VGgUPWdr	Non-specific
U1	PW5100	1	0.04	0.04	https://e.tb.cn/h.6o5VB7iqtapEZrm	Non-specific
U2	XC6206P332MR	1	0.01	0.01	https://e.tb.cn/h.6o3TegHxri4ZuHx	Non-specific
U3	TP4057	1	0.03	0.03	https://e.tb.cn/h.6o5SCsF8ZZHXkyQ	Non-specific
U4	DX-BT24-S	1	1.52	1.52	https://e.tb.cn/h.6o5ReNe95MPK0iJ	Non-specific
U5	STM32L431CCT6	1	1.65	1.65	https://e.tb.cn/h.6o5lKw6qavM0bDQ	Non-specific
U6	AD8232ACPZ-RL	1	1.72	1.72	https://e.tb.cn/h.6o5nESHSxtiMnwe	Non-specific
U7	TPS60403DBVR	2	0.03	0.06	https://e.tb.cn/h.6o5pmM63swd0CNw	Non-specific
U8	AD8642ARZ	2	1.5	1.5	https://e.tb.cn/h.6oU60zpQoaGLEqS	Non-specific
USB1	TYPE-C-6P	1	0.02	0.02	https://e.tb.cn/h.6oU7Ts75W8BAM5z	Non-specific
X1	32.768KHz	1	0.03	0.03	https://e.tb.cn/h.6o0Ci4HvIrYZJHV	Non-specific
X2	25 MHz	1	0.1	0.21	https://e.tb.cn/h.6o3wHlqwl0PF5Is	Non-specific
Battery	70 mA	1	0.75	0.75	https://e.tb.cn/h.6o16f4HAjQdaIoy	Electronics
Total	−	−	−	9.20	−	−

**Table 3:** Mechanical components form.

**Designator**	**Component**	**Number**	**Cost per unit-USD**	**Total cost** **−USD**	**Source of materials**	**Material type**
Lid	3D Printed	1	0.49	0.49	http://myc3d.com/	resin
Enclosure	3D Printed	1	1.58	1.58	http://myc3d.com/	resin
Flat Head Screw	M2*8	4	0.01	0.04	https://e.tb.cn/h.6ogAMxiTIBlBUmB	304 stainless steel
Flat Head Screw	M2*6	4	0.01	0.04	https://e.tb.cn/h.6ogAMxiTIBlBUmB	304 stainless steel
Hexagonal Copper Column	M2*11	4	0.01	0.04	https://e.tb.cn/h.6o4Y96oIuIYuf6s	Copper
Hexagonal Copper Column	M2*10 + 3	4	0.01	0.04	https://e.tb.cn/h.6o4Y96oIuIYuf6s	copper
Shielding Wire	4 core, Length: 1 M	1	0.24	0.24	https://e.tb.cn/h.6op4gxZ5TSy7kFR	Non-specific
Rubber Shell Plug	XH2.54-5P	1	0.01	0.01	https://e.tb.cn/h.6oRkw11DfCclUBL	Non-specific
Chest Strap	Length: 1 M	1	1.9	1.79	https://e.tb.cn/h.6oH4cylukErZTat	Non-specific
Connector Terminal Spring	XH2.54	5	0.01	0.05	https://e.tb.cn/h.6oRydsE4Jb9z2hp	Non-specific
Total	−	−	−	4.32	−	−

## 5 Build instructions

### 5.1 Manufacturing the main control board

**Fig. 5:**
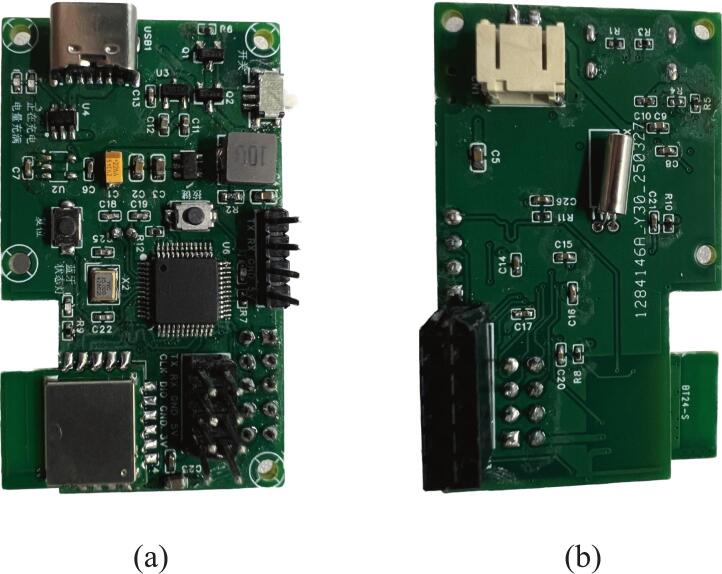
Photograph of the assembled main control board. (a) Front view; (b) Back view.

The manufacturing process of the main control board includes critical steps such as circuit design, PCB fabrication, soldering components, overall assembly, and final functional testing. The PCB is fabricated from a four-layer FR-4 substrate to enhance signal integrity and noise immunity. It is manufactured by Shenzhen JLC Technology Group Co., Ltd., ensuring consistent quality and precision. The components listed in Table 2 and the design specifications are carefully soldered onto the main control board. Strict temperature and timing controls during soldering ensure reliable joints, optimal electrical performance, and increased durability. The assembled main control board is shown in Fig. 5.

### 5.2 Manufacturing of the AFE board

**Fig. 6:**
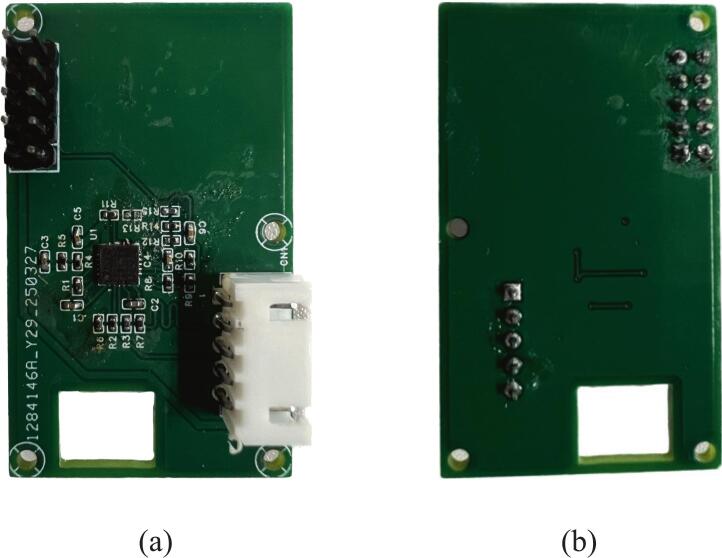
Photograph of the assembled AFE board. (a) Front view; (b) Back view.

To ensure structural integrity and stable signal quality, the AFE board is designed using a four-layer FR-4 substrate, similar to the main control board. The manufacturing process is similar to that of the main control board and includes PCB fabrication and precise soldering of components. Fig. 6 shows photographs of the assembled AFE board displaying both the front and back.

### 5.3 Manufacturing of the dry active electrodes

**Fig. 7:**
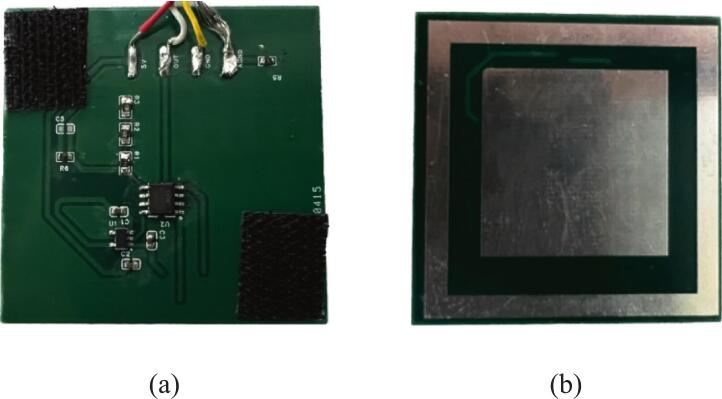
Photograph of the assembled dry electrodes. (a) Front view; (b) Back view.

The dry active electrodes were fabricated using a four-layer FR-4 copper-clad board with a total thickness of 1.6 mm and conventional printed circuit board manufacturing processes [32]. Each electrode integrates an active voltage follower circuit with AD8642 amplifiers, as well as resistors as described in Section 2.1. Two such electrodes were produced to sense non-contact ECG signals and provide stable performance and noise immunity. The fabrication process follows the same standard PCB manufacturing procedures used for the other system circuit components. To ensure secure installation and ease of wear, two pieces of Velcro, each measuring approximately 10 × 10 mm^2^, are diagonally attached to each electrode plate. This allows the electrodes to be firmly mounted onto the chest strap, ensuring stable positioning and consistent signal acquisition. Fig. 7 shows the assembled dry electrodes.

### 5.4 Non-contact ECG system assembly

**Fig. 8:**
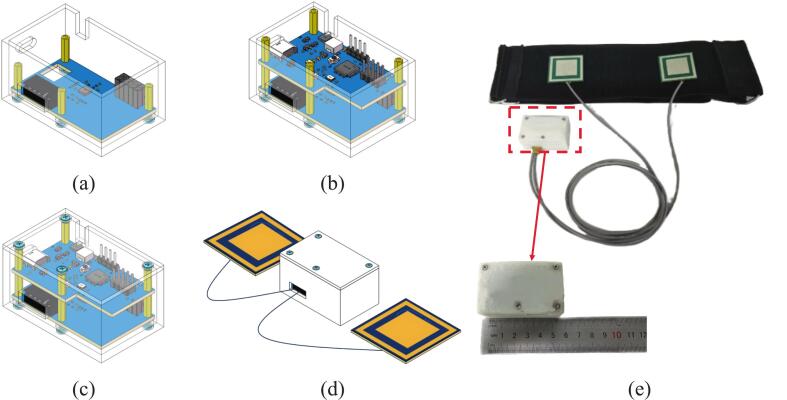
Assembly steps of the proposed non-contact ECG system. (a) Installation of the AFE board into the enclosure base; (b) Installation of the main controller board; (c) Attachment of the top cover of the enclosure; (d) Final assembled 3D model of the ECG device. (e) Prototype of the assembled non-contact ECG device.

Begin by soldering four shielded wires to each electrode board in the following order: power positive, signal output, power ground, and metal shielding. Next, connect the power positive, ground, and shielding wires from both electrode boards together, while keeping the signal output wires separate to avoid cross-interference. After completing these connections, crimp the five resulting wires into terminal pins and insert them into an XH2.54-5P connector to finalize the wiring interface. The pin definitions of the XH2.54-5P connector (CN1) are shown in Fig. 3(b). Next, mount the AFE board inside the enclosure using 11 mm copper standoffs and M2 screws, aligning it with the designated mounting holes. Connect the lithium battery to the PH2.0 connector located on the back of the main control board. Then, position the main control board above the AFE board and secure it with M2 × 10 + 3 mm male brass standoffs and screws. Finally, secure the enclosure's top cover with M2 screws and insert the electrode module connector into the corresponding XH2.54 socket on the AFE board. These assembly steps are visually illustrated in Fig. 8 (a)∼(d). The fully assembled prototype is shown in Fig. 8(e). The finished assembled device is compact, measuring 50 × 35 × 28  mm3 and weighing approximately 60  g.

## 6 Operation instructions

**Fig. 9:**
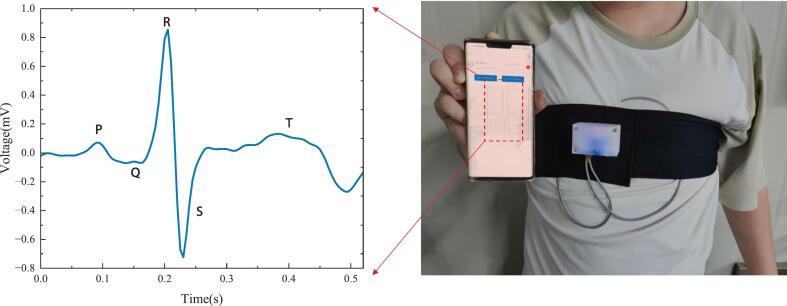
Photograph of the non-contact ECG system in actual use during cardiac monitoring.

First, wear the fully assembled device on your chest and gently tighten the chest strap to ensure a secure fit. Position the electrodes accurately at the standard single-lead locations, firmly over smooth clothing, making sure there is no direct skin contact. Proper placement is crucial for stable and accurate signal acquisition. Fig. 15 shows the recommended electrode positions (yellow squares), and Fig. 9 displays the device in actual use. Once the electrodes are in place, turn on the device. The indicator light will begin flashing, signaling that the device has entered Bluetooth discoverable mode.

**Fig. 10:**
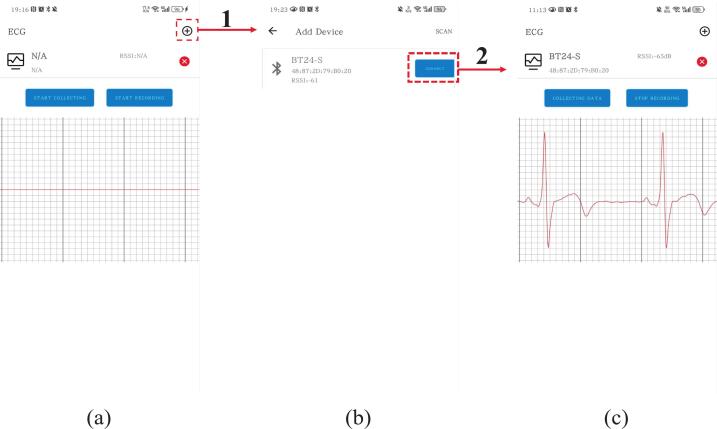
User interface of the non-contact ECG monitoring mobile app. (a) Graphic user interface. (b) Bluetooth scanning and the ECG device connection interface. (b) Real-time ECG signal display and control interface.

Next, open the non-contact ECG monitoring mobile app. As shown in Fig. 10(a), tap the Bluetooth icon in the top right corner to access the device scanning interface. The app will automatically search for nearby Bluetooth devices. If the desired device is not found, tap the “Scan” button in the upper-right corner to retry. Once the target device (BT24-S) appears in the list, tap “Connect”, as shown in Fig. 10(b). Upon successful connection, the app will return to the main interface, as shown in Fig. 10(c). The interface will now display the device name, MAC address, and signal strength (RSSI). The “Start Collecting” button will automatically change to “Collecting Data”, indicating that real-time ECG acquisition has begun. The central area of the screen will display a scrolling ECG waveform in real time, allowing you to visually monitor cardiac activity. The app also features a recording button that toggles between “Start Recording” and “Stop Recording”. Pressing this button saves the current ECG waveform data locally on the mobile device, enabling offline analysis or review.

Device setup, app operation, and real-time ECG monitoring are also demonstrated in the tutorial video, which is provided alongside the source code and design files on the Zenodo open-source platform.

## 7 Validation and characterization

To validate the effectiveness and usability of the proposed open-source, non-contact ECG system, we conducted testing experiments that included evaluating the performance of the electrode, signal chain, and power consumption. In addition, we compared ECG signals collected from human volunteers using the proposed system with signals obtained from a commercial wet-electrode-based system Heal Force Prince-180D ECG Monitor (Heal Force Bio-Meditech Holdings Limited). We also conducted a comparison with previously reported non-contact ECG systems.

### 7.1 Evaluation of active dry electrode performance

**Fig. 11:**
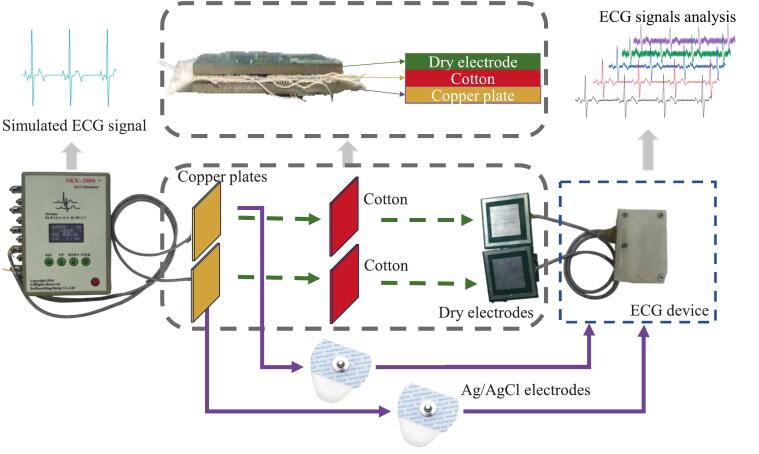
Comparative experimental setup between the proposed non-contact dry electrodes and the commercial wet Ag/AgCl electrodes with our proposed ECG system.

To evaluate the signal acquisition performance of the proposed ECG system and compare the signal quality of non-contact active dry electrodes with that of commercial Ag/AgCl wet electrodes, we set up an experimental setup as shown in Fig. 11. A consistent, simulated ECG waveform was generated using an ECG signal generator (SKX-2000D+, Xuzhou Mingsheng Electronics Co., Ltd.) as the reference input. Our ECG system received signals from the non-contact active dry electrodes and the commercial wet electrodes, respectively. The wet electrodes were directly connected to the signal generator. The dry electrodes were placed on the opposite side of signal-emitting copper plates, and separated by cotton fabric layers of varying thicknesses to simulate different non-contact sensing conditions. To characterize the performance of the active dry electrodes, ECG signals were collected under cotton layers of five different thicknesses: 0.24 mm, 0.72 mm, 1.2 mm, 1.68 mm, and 2.16 mm. A signal-to-noise ratio (SNR) metric was adopted to quantitatively evaluate the signal quality under each condition. The SNR [33] was calculated for each thickness based on Eq. (1)

(1) $$\mathrm{SNR}=10\log(\frac{\sum V_{T}^{2}}{\sum {\left(V_{T}-V_{s}\right)}^{2}})$$

where $V_{T}$ is the ECG waveform extracted using the template drift analysis method, and $V_{S}$ is the recorded signal from the experimental setup.

**Fig. 12:**
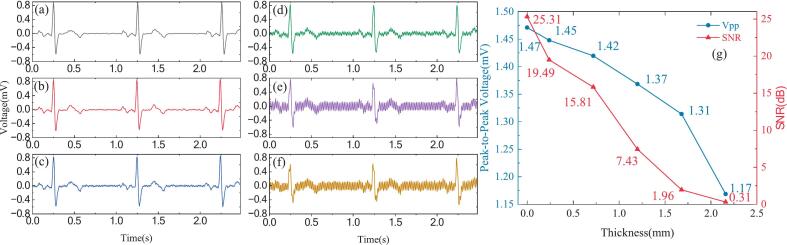
ECG signals acquired using non-contact dry electrodes under varying cotton fabric thicknesses. (a) Commercial Ag/AgCl electrodes with direct contact (0  mm); (b)∼(f) Non-contact dry electrodes with interposed cotton layers of 0.24 mm, 0.72 mm, 1.2 mm, 1.68 mm, and 2.16 mm thickness, respectively; (g) Variation of Vpp and SNR with fabric thickness.

Fig. 12 shows the recorded ECG signals acquired using active dry electrodes under cotton fabric layers of varying thicknesses, and compared with signals obtained from commercial Ag/AgCl wet electrodes in direct contact with the signal source. As illustrated in the figure, are shown in Fig. 12 (a–f), the signal quality was evaluated across different fabric thicknesses. Quantitative metrics including SNR and peak-to-peak voltage (Vpp) are summarized in Fig. 12 (g).

When the cotton layer was thin (e.g., 0.24 mm), the non-contact ECG signals clearly exhibited identifiable P–QRS–T waveforms with minimal noise. As the fabric thickness increased to 0.72 mm and 1.2 mm, there was a gradual reduction in signal amplitude and waveform clarity was observed, though the essential morphological features remained distinguishable. These results indicate acceptable acquisition performance under light-to-moderate clothing conditions. However, with thicker fabric layers of 1.68 mm or 2.16 mm, the signals showed noticeable degradation, including QRS blurring, T wave suppression, and significant power-line interference. The SNR declined sharply from 19.49 dB at 0.24 mm to 0.31 dB at 2.16 mm, and the Vpp dropped from 1.45 mV to 1.17 mV. This performance degradation is primarily due to the increased capacitive impedance and reduced coupling efficiency introduced by thicker dielectric layers. The equivalent capacitance formed between the electrode and the skin depends heavily on the dielectric properties of the intermediate fabric, including its material type, thickness, and contact area [34,35]. As the fabric thickness increases, the capacitive coupling effect diminishes, resulting in signal attenuation and increased noise levels. Therefore, when deploying non-contact ECG systems in practice, especially for ambulatory or long-term monitoring applications, a careful trade-off must be made between user comfort and signal integrity. For our open-source non-contact ECG system, the results indicate that reliable signal acquisition is possible through clothing up to approximately 1.2 mm thick. Beyond this threshold, while the positions of the QRS complexes remain identifiable and the overall rhythm is still discernible, the signal quality deteriorates markedly. This could compromise the accuracy and reliability of continuous monitoring.

**Fig. 13:**
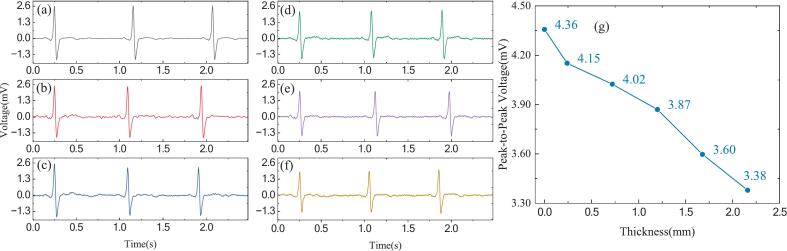
ECG signals acquired from human subjects with different skin-to-electrode fabric thicknesses. (a) ∼(f) Non-contact dry electrodes with interposed cotton layers of 0  mm, 0.24 mm, 0.72 mm, 1.2 mm, 1.68 mm, and 2.16 mm thickness, respectively; (g) Variation of Vpp with fabric thickness.

In addition, we investigated the impact of thickness on signal quality by inserting different layers of fabric between the skin and the electrode during human-subject recordings. As shown in Fig. 13, increasing the thickness of the fabric resulted in a progressive reduction in peak-to-peak signal amplitude, which is consistent with theoretical expectations. Compared to the results in Fig. 12, the signals obtained from the human subjects exhibited lower noise. This can be attributed to two factors. First, the intrinsic amplitude of physiological ECG signals is generally higher than that of simulated signals generated by the SKX-2000D + . Second, skin perspiration can moisten the cotton textile, improving capacitive coupling and enhancing signal quality. We previously explored this mechanism in our electrode design work [35]. Overall, these findings demonstrate that the proposed active dry electrode meets the requirements for reliable ECG monitoring.

### 7.2 Evaluation of signal chain performance

Fig. 9 also shows the ECG waveform acquired using our system while a volunteer wore a 0.35-mm-thick cotton T-short. The clear morphology and distinct QRS complexes demonstrate the ability of the proposed system to effectively perform non-contact ECG acquisition under light clothing. These results confirm the practical feasibility and effectiveness of our system in wearable monitoring scenarios.

**Fig. 14:**
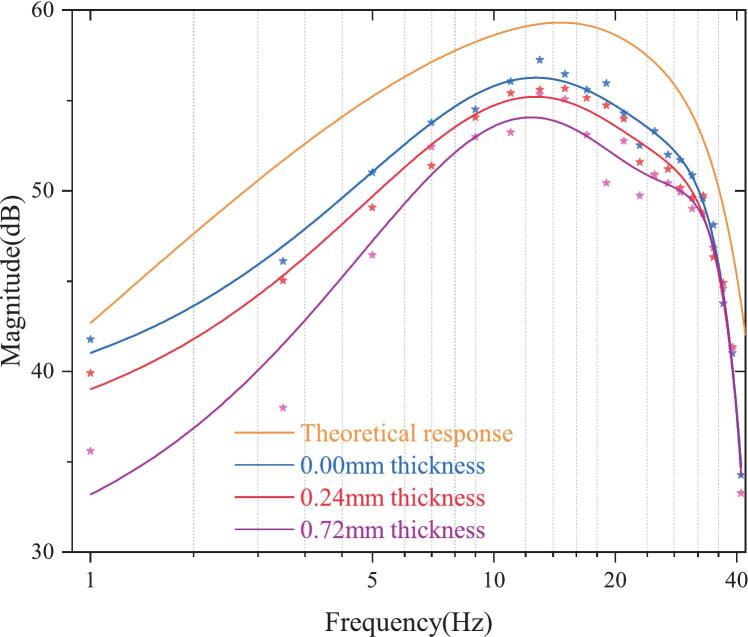
Frequency response of the system under different fabric thicknesses compared to the theoretical model.

Furthermore, we evaluated the signal chain performance. Specifically, we analyzed the frequency response of the signal after passing through the AFE board under three conditions: direct contact and non-contact configurations with cotton fabric of thicknesses 0.24 mm and 0.72 mm between the signal source and the electrode. The experimental results are shown in Fig. 14.

Fig. 14 shows the amplitude-frequency responses of our system under different conditions. The yellow curve represents the theoretical transfer function, and the blue curve shows the measured response with direct-contact (0 mm fabric thickness). Although the designed AFE board has a theoretical gain of approximately 1100, the actual measured gain is slightly lower due to the roll-off effects introduced by the filtering stages [30]. Nevertheless, the close alignment between the measured and theoretical curves confirms that the AFE board exhibits the expected frequency-domain behavior under perfect coupling conditions. To further examine the effect of fabric-based capacitive coupling feature, we evaluated the system frequency response with 0.24 mm and 0.72 mm cotton layers placed between the electrode and the signal-emitting surface. The results (red and purple curves) show a significant decrease in gain, especially within the 1–30 Hz range. Attenuation becomes more pronounced as the fabric thickness increases. These results confirm that clothing acts as a high-impedance dielectric layer when interposed, increasing capacitive reactance, weakening coupling, and degrading biopotential transmission, particularly in the low-frequency range. Future improvements should focus on optimizing the AFE and implementing compensation strategies to mitigate signal degradation caused by clothing-induced impedance.

### 7.3 Power consumption of system

To evaluate the energy efficiency of the proposed non-contact ECG system, the average operating current was measured using an ammeter. Then, the total power consumption was calculated using Eq (2), where U is the supply voltage and I is the measured current.

(2) P=U·I

To analyze the power distribution among the different modules, each component was tested individually. First, the signal conditioning and electrode boards were disconnected, and the main control board was powered on. With the MCU and Bluetooth modules in sleep mode, the baseline power of the power supply module was recorded as 0.84 mW. Then, the MCU (STM32) and the Bluetooth module (DX-BT24-S) were activated sequentially. They contributed 4.28 mW and 3.45 mW (in active mode) or 3.21 mW (in standby mode), respectively. After reconnecting the signal conditioning board, the AD8232 analog front end consumed 0.56 mW. Finally, the electrode board with the AD8642 consumed a total of 9.66 mW, 5.37 mW of which was attributed to the charge pump circuitry that provided the dual supply voltage for the AD8642. The total power consumption of the system was approximately 24.16 mW under normal operating conditions. This modular assessment provides a detailed understanding of the power distribution across the system and serves as a basis for future power optimization in wearable and long-term ECG monitoring applications.

### 7.4 Comparison studies

**Fig. 15:**
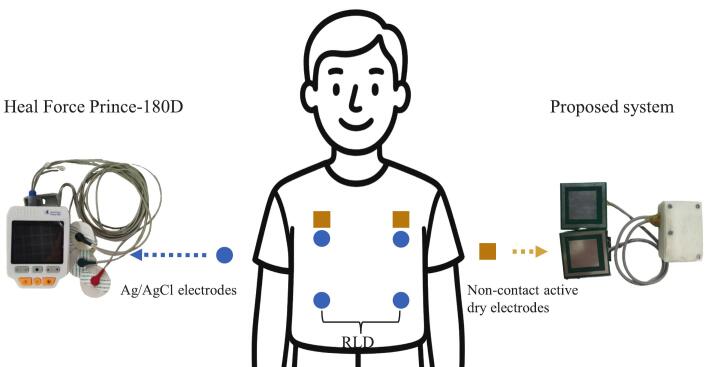
Electrode placement diagram for signal comparison between commercial wet electrodes (blue circles) and proposed non-contact dry electrodes (yellow squares). (For interpretation of the references to colour in this figure legend, the reader is referred to the web version of this article.)

A comparative analysis was performed to evaluate the consistency and accuracy of the proposed non-contact ECG system against a conventional device using Ag/AgCl wet electrodes. ECG signals were simultaneously recorded from a volunteer using both the proposed non-contact active dry electrode system and a commercial reference device, the Prince-180D ECG monitor (Heal Force Bio-Meditech Holdings Limited). To ensure signal consistency and minimize spatial variability, the Ag/AgCl electrodes were placed as close as possible to the capacitive dry electrodes. This enabled both systems to acquire signals from nearly identical anatomical locations, as illustrated in Fig. 15. While the Ag/AgCl electrodes were attached directly to the skin, the proposed dry electrodes acquired signals through a layer of clothing.

**Fig. 16:**
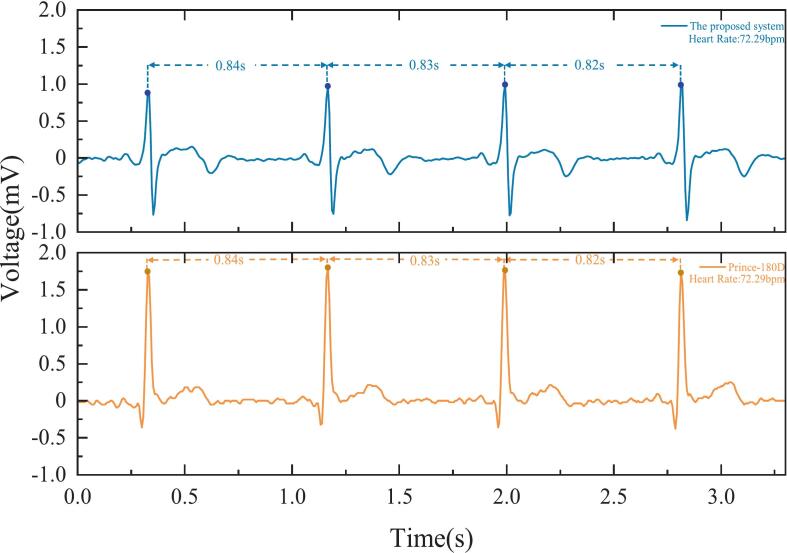
Comparison of ECG waveforms acquired using Prince-180D with wet electrodes and the proposed non-contact ECG system.

Fig. 16 shows the ECG waveforms acquired simultaneously by the proposed non-contact system and the reference Prince-180D monitor. Both systems captured clear P-QRS-T structures with high signal consistency overall in heart rate and R-peak timing. The interbeat intervals measured by the proposed system were 0.84, 0.83, and 0.82 s, which closely matched the intervals measured by the Prince-180D (0.84, 0.83, and 0.82 s). This indicates excellent temporal alignment and heart rate estimation accuracy, as both systems show a heart rate of ∼ 72.29 beats per minute. These results validate the capability of the proposed system to reliably detect cardiac rhythm. However, differences in waveform morphology are also evident. These variations can be attributed to multiple factors. First, the electrode–skin interface differs significantly between the two systems. The proposed device uses capacitive coupling through clothing, which introduces higher impedance and frequency-dependent attenuation. In contrast, the Prince-180D uses Ag/AgCl wet electrodes that are in direct contact with the skin, resulting in a low-impedance interface. Second, differences in system filter bandwidths also contribute to waveform discrepancies. The proposed system uses a 1–40 Hz bandpass filter optimized for the key spectral range of ECG signals. In contract, the Prince-180D’s filter configuration is undisclosed, but may use a wider passband. This difference may influence influencing waveform sharpness. Third, the presence of a dielectric layer (i.e., clothing) in the non-contact system introduces additional capacitive reactance, altering the signal’s frequency response and impacting amplitude and morphology. Despite these differences, both systems exhibit consistent inter-beat intervals and rhythm detection, confirming the reliability of the proposed system for continuous ECG monitoring. A demonstration video showcasing the real-time performance of the non-contact ECG system is available at https://zenodo.org/records/15765815.

**Fig. 17:**
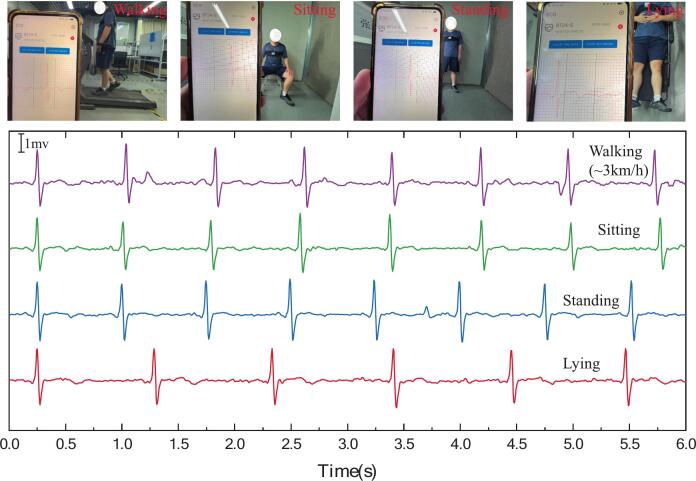
ECG waveforms acquired under various postures and activities (walking, sitting, standing, lying) using the proposed non-contact ECG system.

To further address the challenges of dynamic ECG acquisition in wearable applications, we performed additional validation monitoring real-time signals from human subjects. As shown in Fig. 17, the proposed system reliably captures ECG waveforms across various body postures and motion states (walking, sitting, standing, and lying). The system maintains beat-to-beat consistency and captures clear QRS complexes even during ambulatory motion, demonstrating strong robustness against motion artifacts.

To provide broader context and evaluate the performance of our system, we conducted a comparative study. Table 4 summarizes representative single-lead non-contact ECG systems that employ various electrode designs, configurations, and performance characteristics. Key parameters include electrode flexibility, size, placement, use of right-leg drive (RLD), wireless capability, open-source availability, and SNR.

**Table 4:** Comparison of single-lead non-contact ECG systems with different electrode types, sizes, configurations, and performance.

**Ref.**	**Electrode characteristics**			**Open-source**	**RLD**	**Wireless**	**SNR (dB)**
	Flexible	Size (mm^2^)	Position				
Guermandi [36]	No	78.5	Chest	No	No	BLE	N.a.
Masihi [6]	Yes	706.86	Chest	No	No	BLE/WiFi	23.1
Wang [37]	Yes	7200	Chest	No	Yes	No	29.8
Zheng [33]	Yes	N.a.	Back	No	Yes	No	26.8
Zhou [38]	Yes	1728	Chest	No	Yes	No	N.a.
Proposed	No	2500	Chest	Yes	No	BLE	19.5

Most of the systems listed in Table 4 adopt flexible electrodes to improve wearability and better conform to body surfaces, as demonstrated in references [6,33,37,38]. In contrast, our proposed system uses rigid electrodes and still achieves a competitive SNR of 19.5 dB. Notably, Wang [37] reports the highest SNR of 29.8 dB, but this system lacks wireless capability and open-source accessibility. Masihi [6] achieves an SNR of 23.1 dB with flexible electrodes and BLE/WiFi support, while Zheng [33] obtains 26.8 dB using back-mounted electrodes without wireless communication. The electrode size among these systems ranges widely from 78.5 mm^2^ to 7200 mm^2^. However, the results show that a larger electrode area does not directly translate to better signal quality. Instead, factors such as electrode design, dielectric properties, and circuit architecture play a more decisive role [39]. One key reason our system does not lead in SNR performance is the absence of an RLD circuit, similar to that in [6], which is crucial for enhancing common-mode rejection and reducing power-line interference. Future iterations will incorporate an RLD circuit to improve signal robustness and quality. Importantly, unlike most existing systems, which are that are either proprietary or lack technical transparency, our system is fully open source and provides documentation for both the hardware and software. This transparency is especially valuable for non-contact ECG research, where system integration, particularly AFE and active electrode design, poses substantial technical challenges.

Our platform offers a practical and replicable foundation for non-contact ECG monitoring. Currently, it is best suited for wearable applications, such as heart rate and rhythm tracking, rather than clinical-grade diagnostics. Future work will focus on integrating flexible electrodes and incorporating an RLD circuit to further improve user comfort and signal quality. The active electrode circuit will also be optimized with an emphasis on low-power design. As reported in Section 7.3, the AD8642 amplifier alone accounts for approximately 9.66 mW, about 40 % of the total system power. This poses a notable limitation for long-term, battery-powered operation. This highlights the importance of improving energy efficiency in future iterations. In addition, improving ECG waveform fidelity in non-contact configurations remains a significant challenge. Meeting clinical diagnostic requirements by bridging this gap will be a key direction for future research and system refinement.

## 8 Conclusion

In summary, we have developed and validated a miniaturized, fully open-source non-contact ECG monitoring platform based on active dry electrodes. The 50 × 35 × 28 mm^3^ hardware node weighs 60 g and costs approximately $14. It integrates ultra-high-impedance active electrodes, an analog front-end for signal amplification and filtering, efficient power management, and Bluetooth-enabled wireless data transmission to a mobile application. A comprehensive evaluation demonstrates that the device can reliably acquire ECG signals through clothing. Key rhythm features, such as QRS complexes, are clearly preserved, and heart rate is accurately estimated. Comparative studies confirm temporal consistency with clinical-grade wet electrode monitors, despite inherent differences in signal morphology due to coupling and filtering effects. Systematic power consumption analysis also identified opportunities for further optimization, particularly within the active electrode module.

Although the current design uses rigid electrodes and lacks an RLD circuit, our open-source platform offers the community a transparent and replicable reference for hardware and software. Future work will focus on integrating flexible electrodes and RLD circuits to improve comfort, robustness, and noise immunity. In addition, we will work on low-power circuit design to enable extended wearable operation. Releasing all design files and firmware openly aims to accelerate innovation and collaboration in the field, making advanced non-contact ECG monitoring accessible to a wider range of research and application scenarios.

## CRediT authorship contribution statement

**Siluo Chen:** Writing – original draft, Software, Methodology, Data curation. **Yu Chen:** Writing – review & editing, Writing – original draft, Validation, Methodology. **Jing Huang:** Writing – review & editing, Validation, Supervision. **Chengyu Liu:** Writing – review & editing, Validation, Methodology, Conceptualization. **Kan Luo:** Writing – review & editing, Writing – original draft, Supervision, Software, Project administration, Methodology, Conceptualization.

## Declaration of competing interest

The authors declare that they have no known competing financial interests or personal relationships that could have appeared to influence the work reported in this paper.
